# Clinical significance and antifungal susceptibility profile of 103 clinical isolates of *Scedosporium* species complex and *Lomentospora prolificans* obtained from NIH patients

**DOI:** 10.1128/jcm.01550-24

**Published:** 2025-03-07

**Authors:** Mary M. Czech, Jennifer Cuellar-Rodriguez, Kyung J. Kwon-Chung, Frida Stock, Chioma I. Aneke, Kenneth N. Olivier, Kevin P. Fennelly, Juan Gea-Banacloche, Christa S. Zerbe, Alexandra F. Freeman, Steven M. Holland, Michail S. Lionakis, Amir Seyedmousavi

**Affiliations:** 1 Laboratory of Clinical Immunology and Microbiology, National Institute of Allergy and Infectious Diseases, National Institutes of Health 573242 , Bethesda, Maryland, USA; 2 Microbiology Service, Department of Laboratory Medicine, Clinical Center, National Institutes of Health 378162 , Bethesda, Maryland, USA; 3 Division of Pulmonary Diseases and Critical Care Medicine, Department of Medicine, University of North Carolina 214908 , Chapel Hill, North Carolina, USA; 4 Pulmonary Clinical Medicine Section, Cardiovascular Pulmonary Branch, Division of Intramural Research, National Heart, Lung, and Blood Institute, National Institutes of Health 369963 , Bethesda, Maryland, USA; University of Calgary, Calgary, Alberta, Canada

**Keywords:** *Scedosporium *species, *Lomentospora prolificans*, antifungal susceptibility testing, clinical outcomes

## Abstract

Reduced susceptibility to antifungals is common among members of genera *Scedosporium* and *Lomentospora*, with optimal treatments still not fully defined. *In vitro* antifungal susceptibility results and clinical data do not comprehensively account for the advent of new *Scedosporium* species identified by molecular phylogenetics. Using Clinical and Laboratory Standards Institute (CLSI) methodology, we tested a total of 103 clinical isolates obtained from patients at the NIH Clinical Center. The most frequent species were *Scedosporium apiospermum* (63%) and *Scedosporium boydii* (11%), followed by *Lomentospora prolificans* (7%). The novel antifungal olorofim showed the lowest MICs against all *Scedosporium* spp. and *L. prolificans*, followed by micafungin. Among the triazoles, voriconazole showed lower MICs against *Scedosporium* spp. Amphotericin B and posaconazole demonstrated species-specific and inter-species variable activity. Itraconazole, isavuconazole, and terbinafine had higher MIC values against *Scedosporium* spp. and *L. prolificans*. Clinical data were retrospectively reviewed for 90 isolates, of which nine patients (28 isolates) had active disease/infection and received antifungal treatment that included voriconazole or posaconazole. Five of these patients (56%) died, while three patients (33%) with chronic granulomatous disease were cured following hematopoietic cell transplantation. In 24 patients (62 isolates), the presence of the fungus was considered airway colonization. In conclusion, our data support the existence of species-specific and inter-species differences in the antifungal susceptibility patterns among members of genera *Scedosporium* and *L. prolificans*. The novel investigational antifungal olorofim may be a promising therapy. Our clinical data suggest that host status and administration of antifungal therapy most effective for each *Scedosporium* species complex are important determinants of outcomes.

**IMPORTANCE:**

Understanding the epidemiology and clinical spectrum of infections caused by *Scedosporium* species complex and *Lomentospora prolificans* is integral to improving outcomes, particularly in severely ill and immunocompromised patients. *In vitro* antifungal susceptibility testing can provide an estimate of antifungal activity against fungal pathogens. Our study showed that species-specific and inter-species differences exist in the distribution of antifungal susceptibility patterns between *Scedosporium* and *L. prolificans*. Our clinical data also highlight that host status, along with effective antifungal therapy, plays a crucial role in determining treatment outcomes.

## INTRODUCTION

Members of the fungal genera *Scedosporium* and *Lomentospora* have been increasingly recognized as emerging opportunistic pathogens (1). The taxonomy of these two genera has been subject to several revisions on the basis of molecular phylogenetic studies. *Scedosporium prolificans* has been now re-classified to its original name as *Lomentospora prolificans* (2). Excluding cases of aspergillosis, surveillance data indicate that scedosporiosis and lomentosporiosis account for up to 33% of invasive mold infections, depending on the geographic location and patient population (1). *Scedosporium* and *Lomentospora* are environmental molds commonly found in soil, animal droppings, water sources, and sewage (3). As is true for most invasive fungal infections, disease occurs following inhalation or inoculation at cutaneous or mucosal barriers (4). Clinical manifestations range from superficial infections (5) and colonization of the respiratory tract (6, 7) to eumycetoma (8), severe invasive localized or disseminated disease (9, 10), and fungemia (11). Disease most commonly develops in immunocompromised hosts, although infections have also been reported in previously healthy individuals (12). Patients with disorders of neutrophils and/or T-cell dysfunction, such as those with hematologic malignancies and recipients of solid organ or hematopoietic stem cell transplantation, are at increased risk for severe disease with poor prognosis (13, 14).

According to retrospective studies and evidence-based guidelines, voriconazole (VRC) monotherapy is considered the primary choice of treatment for *Scedosporium* and *Lomentospora* infections, but combination therapy with terbinafine is frequently recommended for better outcomes, especially in patients with disseminated infections (15). There are no established breakpoints or epidemiologic cut-off values for *Scedosporium* spp. and *L. prolificans*. Susceptibility data for clinical isolates, although limited, tend to show elevated minimum inhibitory/effective concentrations (MICs/MECs) and do not comprehensively account for the advent of new *Scedosporium* spp. established by molecular phylogenetics from the early 2000s to the present (16). Updated *in vitro* drug susceptibility and clinical data are needed to address these knowledge gaps.

Herein, we characterized the species identification and *in vitro* antifungal susceptibility profiles of a large number of *Scedosporium* and *Lomentospora* clinical isolates and correlated our *in vitro* data with clinical parameters and outcomes. Our study is among the largest to provide both *in vitro* antifungal susceptibility and clinical data of *Scedosporium* and *Lomentospora* isolates.

## MATERIALS AND METHODS

### Setting and epidemiological data

Fungal isolates and retrospective clinical data were collected from patients seen at the National Institutes of Health (NIH) Clinical Center, Bethesda, Maryland, USA. The NIH Clinical Center is a 200-bed research hospital that serves patients from geographically diverse areas, including both domestic and international sites.

### Fungal isolates

A total of 103 clinical isolates identified as *Scedosporium* or *Lomentospora* were collected from 1 January 2013 to 31 December 2022. Initial species-level identification of each isolate was attempted using colony morphology, microscopic characteristics, and matrix-assisted laser desorption/ionization time-of-flight mass spectrometry (MALDI-TOF Biotyper Bruker Daltonics Inc. Billerica, MA, USA). The species identity of each isolate was then confirmed via PCR amplification and sequence-based analysis of the internal transcribed spacer (ITS) region of the ribosomal DNA (rDNA) and the calmodulin gene, as previously described (2).

### Antifungal susceptibility testing

Antifungal susceptibility testing was conducted in accordance with the Clinical and Laboratory Standards Institute (CLSI) M38-A3 guidelines (17). For all drugs except micafungin, the minimum inhibitory concentration (MIC) was defined as the lowest concentration that completely inhibited growth as assessed by visual inspection in comparison with the control (drug-free well). For micafungin, the minimum effective concentration (MEC) was defined as the lowest concentration at which abnormal, short, and branched hyphal clusters were observed, as opposed to the long, unbranched hyphal elements seen in the control well. The densities of the conidial suspensions used for antifungal susceptibility testing, the concentrations of antifungals tested, and incubation times for determining MIC and MEC are summarized in Table S2. Olorofim (OLF) was provided by F2G, Ltd. (Manchester, United Kingdom), and all other drugs were purchased from Sigma (St. Louis, MO).

To ensure the reliability and reproducibility of results, *Candida krusei* (ATCC 6258), *Aspergillus fumigatus* (ATCC MYA-3626), *Scedosporium apiospermum* (ATCC MYA-3635), and *Trichophyton mentagrophytes* (MRL1957, ATCC MYA-44394) were used as quality control strains on each day of testing. Each experiment was performed in three independent replicates on different days.

### Data analysis

The geometric mean (GM), range, and MIC/MEC 50/90 distributions were analyzed using GraphPad Prism, version 9.0, for Windows (GraphPad Software, San Diego, CA).

### Clinical data

Medical charts were retrospectively reviewed to collect clinical data. Patients were classified as having *Scedosporium* spp. or *L. prolificans* “colonization”, “disease”, or “infection” defined as below:

1. Patients were classified as having “colonization*”* if antifungal therapy was not administered for the purposes of treating *Scedosporium* spp. or *L. prolificans*, and clinical symptoms/radiographic features remained stable, or an exacerbation of clinical symptoms was attributed to an alternative etiology.
2. Patients were classified as having *definite, probable,* or *possible invasive* fungal “disease” due to *Scedosporium* spp. or *L. prolificans* based on definitions outlined by the European Organization for Research and Treatment of Cancer and the Mycoses Study Group Education and Research Consortium (18).
3. Patients were classified as having “infection*”* if:
  - Diagnostic criteria for “disease*”* were not met (e.g., patients were not classically immunocompromised hosts);
  - AND there was concern for invasive fungal disease based on clinical symptoms, radiographic findings, and/or culture data OR there was concern for allergic bronchopulmonary mycosis or other suspected inflammatory effects due to *Scedosporium* spp. or *L. prolificans*;
  - AND systemic antifungal therapy was administered for the purposes of treating *Scedosporium* spp. or *L. prolificans*.

## RESULTS

### Distribution of fungal isolates

**Fig 1:**
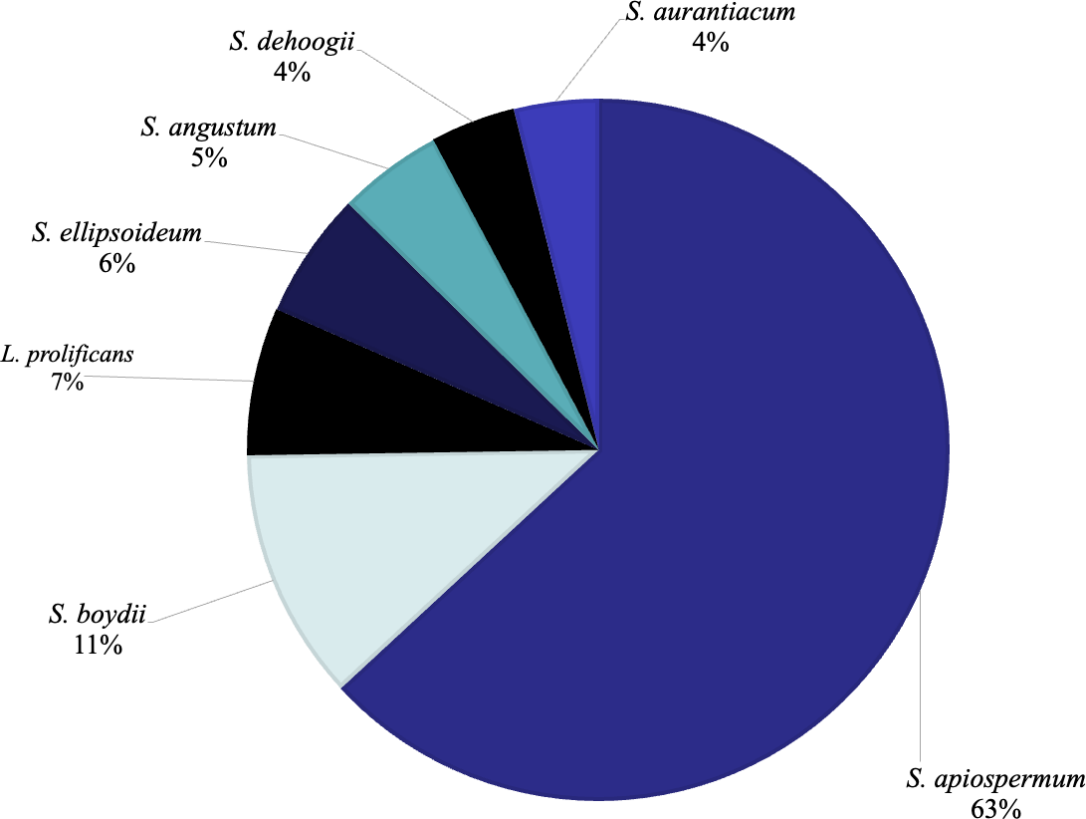
Distribution of molecularly identified *Scedosporium* spp. and *Lomentospora prolificans* isolates during the period 2013–2022.

The frequency of *Scedosporium* spp. and *L. prolificans* isolates are detailed in Fig. 1. The most common isolates were *Scedosporium apiospermum* (63%), followed by *S. boydii* (11%), and *L. prolificans* (7%). *Scedosporium ellipsoideum* (6%), *S. angustum* (5%), *S. dehoogii* (4%), and *S. aurantiacum* (4%) comprised the minority of isolates.

### Antifungal susceptibility data

*In vitro* susceptibility results of eight antifungals against *Scedosporium* spp. and *L. prolificans* isolates are listed in Table 1; Table S1. The novel antifungal OLF showed the lowest MICs against all *Scedosporium* spp. and *L. prolificans*, followed by micafungin (MFG). MFG MECs were similarly low, except for elevated MECs against *S. aurantiacum* and *L. prolificans*, while MECs against *S. ellipsoideum* were variable. Among triazoles, VRC showed the lowest MICs against *Scedosporium* spp. Compared with VRC, posaconazole (POSA) was specifically noted to have higher MICs against *S. ellipsoideum* and *S. aurantiacum*. Amphotericin B (AmB) demonstrated species-specific and inter-species variable activity. Itraconazole, isavuconazole, and terbinafine had high MICs against *Scedosporium* spp. *Lomentospora prolificans* had low MICs for OLF, as indicated above, and otherwise high MIC geometric means with significant inter-species variability for the remaining tested antifungals.

**TABLE 1:** Geometric mean MIC/MEC, MIC/MEC ranges, and MIC/MEC 50/90 for 103 clinical *Scedosporium* spp. and *Lomentospora prolificans* strains to eight antifungal agents^*a*^

Species	# Isolates		MIC/MEC values (µg/mL)							
			AmB	ITC	VRC	POSA	ISA	TRB	MFG	OLF
*S. apiospermum*	65	GM	8.43	9.28	1.14	1.05	6.14	12.07	0.17	0.11
		Range	1–16	0.25–16	1–2	0.125–2	1–16	0.5–16	0.016–0.5	0.031–0.5
		MIC 50	4	16	1	1	8	16	0.125	0.062
		MIC 90	16	16	1.4	2	11.2	16	0.35	0.237
*S. boydii*	12	GM	9.58	10.77	1.08	1.21	7	12.4	0.19	0.12
		Range	1–16	0.25–16	1–2	0.13–2	1–16	0.5–16	0.016–0.5	0.03–0.5
		MIC 50	10	16	1	1.5	8	16	0.18	0.06
		MIC 90	16	16	1	2	15.2	16	0.475	0.46
*S. ellipsoideum*	6	GM	16	16	3.83	6.5	12	16	3.12	0.35
		Range	16	16	1–16	1–16	8–16	16	0.016–16	0.125–1
*S. angustum*	5	GM	1	9.65	1	1.25	5.2	9.62	0.312	0.05
		Range	1	0.125–16	1	0.13–2	1–8	0.06–16	0.031–0.5	0.03–0.06
*S. aurantiacum*	4	GM	14	16	1.5	16	16	16	12.16	4.75
		Range	8–16	16	1–2	16	16	16	0.62–16	0.5–16
*S. dehoogii*	4	GM	5	16	1.25	2.5	7	16	0.37	0.34
		Range	4–8	16	1–2	2–4	4–8	16	0.25–0.5	0.13–0.5
*L. prolificans*	7	GM	11.75	13.79	13.86	13.7	14	16	5.07	0.4
		Range	0.25–16	0.5–16	1–16	0.06–16	2–16	16	0.016–16	0.06–0.5

^*a*^ AmB, amphotericin B; ITC, itraconazole; VRC, voriconazole; POSA, posaconazole; ISA , isavuconazole; TRB, terbinafine; MFG, micafungin; OLF, olorofim; MIC, minimum inhibitory concentration; MEC, minimum effective concentration; GM, geometric mean.

### Clinical data

**Fig 2:**
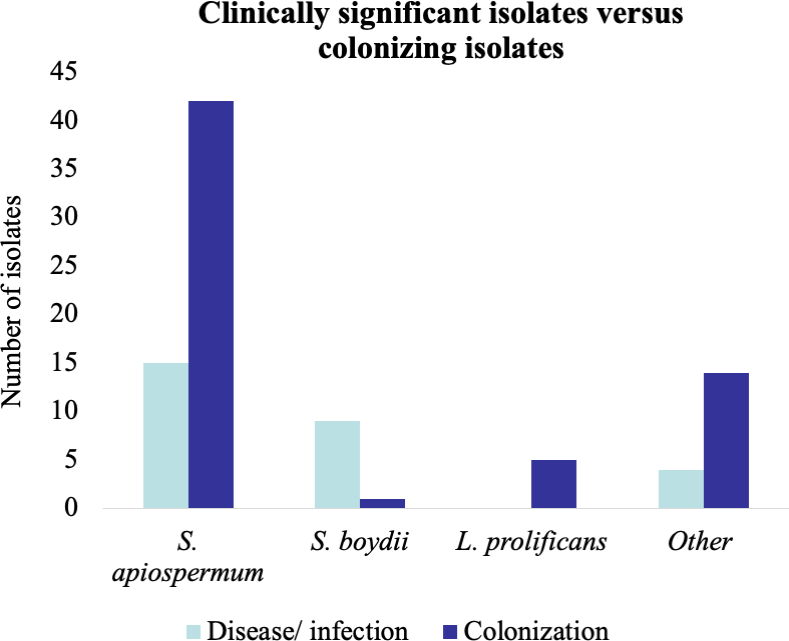
Frequency of clinically significant (disease/infection) versus colonizing *Scedosporium* spp. and *Lomentospora prolificans* isolates among patients hospitalized at the NIH Clinical Center.

Ninety isolates were collected from 33 patients, for whom corresponding clinical data were available for review: 28 isolates were collected from nine patients who had disease or infection with *Scedosporium* spp. or *Lomentospora prolificans*, and 62 isolates were collected from 24 patients who had colonization (Fig. 2). Clinical data were not available for review for the remaining 13 isolates.

Among the nine patients with disease or infection, definite disease was diagnosed in four patients, probable disease in three patients, and infection in two patients (Table 2). Eight out of nine patients were immunocompromised, and one patient had bronchiectasis and pulmonary infection. Notably, two patients had inborn errors of immunity – STAT3-mutated Hyper-IgE syndrome (STAT3-HIES) and autoimmune polyendocrinopathy candidiasis ectodermal dystrophy (APECED) – each leading to underlying bronchiectasis that can make the host susceptible to fungal infections.

**TABLE 2:** Patient data for disease/infection with *Scedosporium* spp.^*a*^

Age, sex, primary diagnosis	Culture source	*Scedosporium* spp.	Classification of disease versus infection	Treatment	Outcome
51 yo M, APECED and pulmonary NTM	Pleural fluid, operative lung biopsy	*S. apiospermum* (nine isolates), *S. angustum* (three isolates), *S. boydii* (one isolate)	Definite pulmonary disease with extension into pleural space	POSA (MIC range 0.25–2), MFG (MEC range 0.125–1), inhaled VRC (MIC 0.5–2)	Died
18 yo M, CGD	Pulmonary fine needle aspirate	*S. apiospermum*	Definite pulmonary disease	POSA (MIC 16)	Died
11 yo M, CGD	Operative lung biopsy	*S. apiospermum*	Definite pulmonary disease	POSA (MIC 0.25), caspofungin (MFG MEC 0.25), TRB (MIC 8)	Cured post-HCT
30 yo M, CGD	Thoracic paravertebral mass	*S. apiospermum*	Definite disease; pulmonary and extension to T2–6 paravertebral space	VRC (MIC 2), caspofungin (MFG MEC 0.25), TRB (MIC 16)	Cured post-HCT
43 yo M, CGD	Bronchoalveolar lavage	*S. dehoogii*	Probable pulmonary disease	POSA (MIC 2), micafungin (MEC 0.5), TRB (MIC 16)	Cured post-HCT
53 yo F, diffuse large B cell lymphoma, receiving EPOCH-R + ibrutinib	Bronchoalveolar lavage	*S. apiospermum*	Probable pulmonary disease	AmB (MIC 16) x10 days with VRC (MIC 1), and then ongoing VRC	Died
37 yo F, Job’s syndrome	Bronchoalveolar lavage	*S. apiosperum*	Probable pulmonary disease	POSA (MIC 1), inhaled VRC (MIC 0.5)	Died
68 yo F, bronchiectasis	Sputum	*S. boydii*	Pulmonary infection	POSA (MIC 0.125–1)	Died
25 yo F, STAT3-HIES	Bronchoalveolar lavage	*S. boydii*	Pulmonary infection with allergic bronchopulmonary process and mycetomas	POSA (MIC 2) and ISA (MIC 8), intermittent TRB (MIC 16), inhaled VRC (MIC 1), Olorofim (MIC 0.062)	Alive—under treatment

^*a*^ APECED, autoimmune polyendocrinopathy candidiasis ectodermal dystrophy; NTM, nontuberculous mycobacteria; CGD, chronic granulomatous disease; EPOCH-R, etoposide, prednisone, vincristine, cyclophosphamide, doxorubicin, rituximab; HCT, hematopoietic cell transplant.

All nine patients had primary pulmonary disease or infection, and one patient had biopsy-proven extra-pulmonary dissemination of *S. apiospermum*. The disease was due to *S. apiospermum* in six cases, of which one case of definite disease yielded additional respiratory cultures with *S. angustum* and *S. boydii*. Another case of disease was due to *S. dehoogii*, and the two cases of infection were due to *S. boydii*. Cultures were monomicrobial for three patients with chronic granulomatous disease (CGD) who had definite pulmonary disease, and otherwise polymicrobial with other fungi and/or bacteria for this group of patients.

All nine patients with disease or infection were treated with a systemic antifungal, which included a triazole. Three patients with CGD were cured of their definite fungal disease with antifungal therapy and hematopoietic stem cell transplantation, which corrected the underlying immunodeficiency. Five patients with disease or infection died, and one patient is still receiving treatment.

Among the 62 *Scedosporium* spp. or *L. prolificans* isolates collected from 24 patients who were colonized with the fungus, the most frequent site of colonization was the respiratory tract (58 isolates). All patients with colonization of the respiratory tract had bronchiectasis. Besides the respiratory tract, colonization was observed in the skin, involving a chronic wound (1 isolate from one patient) and the external auditory canal (3 isolates from one patient). The most common colonizing species was *S. apiospermum* (42 isolates), followed by *L. prolificans* (five isolates), *S. ellipsoideum* (five isolates), *S. aurantiacum* (four isolates), *S. dehoogii* (three isolates), *S. angustum* (two isolates), and *S. boydii* (one isolate). Concurrent with *Scedosporium* spp. or *L. prolificans*, most cultures in colonized patients were polymicrobial (55 cultures). Polymicrobial cultures included a variety of pyogenic bacteria (most commonly *Pseudomonas aeruginosa*), non-tuberculous mycobacteria, and other fungi (most commonly *Aspergillus* spp.).

## DISCUSSION

We present susceptibility and clinical data for a large set of *Scedosporium* spp. and *L. prolificans* clinical isolates. Our results demonstrate significant species-specific and inter-species variability in antifungal activity. OLF, followed by MFG and VRC, tended to have the lowest MICs/MECs. Our clinical data suggest that host status is a critical determinant of treatment outcome. Our findings align with other reports of *Scedosporium* and *L. prolificans* isolate (5, 13). Consistent with previous reports, we found that *S. apiospermum*, followed by *S. boydii* and *L. prolificans*, are among the most frequent clinical isolates (7, 19, 20). We further corroborate species-specific susceptibility data that show inter-species variability (16). Taken together, our data underscore the importance of identifying isolates to the species level and determining antifungal susceptibilities. As a limitation, our data set included multiple isolates of the same species from the same patient. However, this allowed us to make the observation that there was no increase over time in MIC/MEC values of consecutive isolates obtained from the same patient.

The taxonomy and nomenclature of the genera *Scedosporium* and *Lomentospora* have undergone several revisions in the past two decades (12). Currently, 10 species of *Scedosporium* are clinically recognized: *S. aurantiacum*, *S. cereisporum*, *S. dehoogii*, *S. desertorium*, *S. minutisporum*, and *S. apiospermum* species complex that comprises *S. apiospermum sensu stricto*, *S. boydii* (formerly *Pseudallescheria boydii*), *S. angustum*, *S. ellipsoideum*, and *S. fusoideum* (2). In addition, a new species “*Scedosporium americanum”* has been recently reported from a single strain causing a chronic cutaneous infection in a diabetic patient (21). Of note, because of close similarities between spectra of *Scedosporium* species, MALDI-TOF cannot lead to proper species-level identification, and PCR sequencing of multiple targets, including ITS and calmodulin genes, is required. Due to their omnipresence, members of the genus *Scedosporium* are the third most common filamentous fungi isolated from the respiratory tracts of patients with cystic fibrosis (CF), following *Aspergillus* and *Penicillium* (22).

Our study includes a variety of newly identified *Scedosporium* spp., for which previously published *in vitro* data are scant and limited to a small number of isolates. Several of these species are further discussed below.

*S. boydii* is an emerging opportunistic pathogen in immunocompromised hosts, such as those with leukemia, transplantation, or chronic granulomatous disease (CGD), and can produce a wide spectrum of clinical presentations from superficial skin disease and mycetomas to CNS and disseminated disease (23). Moreover, infection in bronchiectasis patients with HIES may be life-threatening, and the proper management and prevention of these infections warrant continued investigation (24).

*S. angustum* and *S. ellipsoideum* are now distinct species within the *S. apiospermum* species complex. *S. angustum* has been reported as a cause of soft tissue infection, corneal abscess, and respiratory infection (21). Our data show that *S. angustum* has a MIC/MEC profile that mirrors that of *S. apiospermum. S. ellipsoideum* has been reported as a cause of soft tissue infection in burn patients (19) and as a respiratory colonizer in CF patients (7). However, compared with *S. apiospermum*, *S. ellipsoideum* tends to have more variable MICs against VRC, POSA, and MFG.

*S. aurantiacum* was established as a distinct species in 2005. It was previously part of the *S. apiospermum* complex, and it may therefore be underdiagnosed. This species has been relatively abundant in environmental and clinical samples in Australia (25, 26), and there has been increasing global recognition of its presence. *S. aurantiacum* has been reported as a cause of corneal ulcer (27), soft tissue infection (21), osteomyelitis (28), brain abscess after near-drowning (29), and respiratory tract colonization in CF patients (20). Compared with other *Scedosporium* spp., *S. aurantiacum* is noted for having variable and elevated MECs/MICs against MFG (GM MEC: 12.16 µg/mL) and OLF (GM MIC: 4.75 µg/mL), raising concerns about suboptimal treatment responses to these drugs. The triazole VRC was, however, shown to be the most active drug *in vitro* (GM MIC, 1.5 µg/mL), which warrants further studies.

*S. dehoogii* was introduced as a species in 2008. It is occasionally found in respiratory samples and has been reported as a cause of corneal abscess (21) and skin/soft tissue infections (30, 31). Our data show that *S. dehoogii* has a MIC/MEC profile similar to *S. apiospermum*.

*L. prolificans* (formerly *Scedosporium prolificans*) is considered distinct from the genus *Scedosporium* based on phylogenetic and morphological differences. *Lomentospora* has only emerged as a severe opportunistic pathogen relatively recently (32), with a wide range of clinical presentations. Disseminated infections are often fatal, particularly in immunocompromised patients with leukemia or transplant recipients. Colonization of CF lungs has been noted, though AIDS-related cases are rare (33).

In general, based on historical susceptibility data and extensive clinical experience, VRC is considered a preferred agent for treating *Scedosporium* spp. (34, 35) and *L. prolificans* (33, 36). Prior reports have shown low MICs for VRC against *Scedosporium* spp. (16, 37, 38), and our data support these findings, although we observed variable MICs among *S. ellipsoideum* isolates. *L. prolificans,* known to be a refractory mold, exhibited variable and elevated MICs to all known antifungals with the exception of OLF (16, 38, 39). Among systemic antifungals, *L. prolificans* is also considered intrinsically resistant to amphotericin B and fluconazole (40).

Historically, patients with scedosporiosis and lomentosporiosis treated with VRC have better clinical outcomes compared with those treated with AmB products (10). However, treatment with VRC for scedosporiosis and lomentosporiosis showed a therapeutic response in only 57% of patients, increasing to 72% when excluding those with major immunosuppression (34). Although *in vitro* synergy testing is not well standardized in clinical microbiology laboratories, there has been an increasing number of case reports describing the successful use of combination antifungal treatment, particularly with VRC plus terbinafine against *L. prolificans* (36). The European Confederation of Medical Mycology (ECMM)/International Society for Human and Animal Mycology (ISHAM)/American Society for Microbiology (ASM) guideline for the diagnosis and management of rare mould infections also strongly recommends VRC plus terbinafine for the treatment of infections caused by *L. prolificans* (15).

The growing population of immunocompromised individuals susceptible to these infections, coupled with suboptimal clinical responses to traditional antifungals, highlights the need for new antifungal treatments targeting resistant mold infections like scedosporiosis and lomentosporiosis. Our data, consistent with other published series, show that OLF has the lowest MICs against *Scedosporium* spp. and *L. prolificans*. Additionally, our study now comprises the largest report of OLF tested against *Scedosporium* spp. isolates (41–45). OLF is a novel antifungal that inhibits the enzyme dihydroorotate dehydrogenase involved in fungal pyrimidine biosynthesis. Despite increasing *in vitro* data, clinical experience with OLF remains limited. *In vivo* mouse models indicate that OLF is effective against *Scedosporium* spp. and *L. prolificans* (46). In humans, the largest reported experience is from a Phase IIb clinical trial, where an interim analysis of 100 patients with difficult-to-treat invasive fungal infections (*n* = 11 with *Scedosporium* spp., *n* = 17 with *L. prolificans*) showed that OLF improved all-cause mortality compared with historic controls who received no therapy or azole, for whom the isolates were known to be resistant (47). The successful overall response was 55%/36% for *Scedosporium* and 53%/53% *L*. *prolificans*. Other clinical experiences with OLF are limited to case reports (48) and an ongoing unpublished Phase III trial for the treatment of Aspergillosis (OASIS), which is still enrolling. Continued accumulation of clinical experience may position OLF as a promising therapy for scedosporiosis and lomentosporiosis.

Our data show MFG also tends to have low MECs against *Scedosporium* spp., except for *S. aurantiacum*, concordant with other reports (16). Echinocandins are not FDA approved or recommended as monotherapy in mold infections due to fungistatic anti-mold activity and poor *in vivo* outcomes in animal mold models (49). However, similar to aspergillosis treatment (50), combination therapy with an echinocandin plus a triazole may have a role in treating *Scedosporium* spp. In murine models, the combination of MFG and a triazole showed a trend toward increased survival in invasive infections with *S. apiospermum* and *S. boydii*, although it did not reach statistical significance (51). Clinical experience is limited (52). Thus, further data are needed to help clinicians optimize treatment regimens.

Our clinical data continue to underscore the importance of host status as a determinant of mold infection. Based on our patient population, most *Scedosporium* spp. and *L. prolificans* isolates were collected from patients with bronchiectasis and respiratory colonization. These patients were not prescribed systemic antifungals, and there was no appreciable impact on clinical outcomes. A smaller subset of patients had disease or infection with *Scedosporium* spp. These patients all received antifungal therapy that included an agent with low MICs/MECs against the cultured isolate of *Scedosporium* spp. The GM and range of MIC/MEC values for these antifungals are summarized in Table 2. However, regardless of antifungal therapy, clinical cure correlated with correction of the underlying immune deficiency that predisposed to the infection.

Effective treatment of scedosporiosis and lomentosporiosis requires both optimizing host status and using effective antifungals. Future research is needed to investigate the synergy of combination therapies, especially involving echinocandins and triazoles. Early *in vitro* data and retrospective chart review suggest that terbinafine plus VRC may improve treatment outcomes for lomentosporiosis (36, 39), but more clinical data are needed.

Emerging classes of antifungals with novel mechanisms of action warrant further study. In addition to OLF, early *in vitro* data on fosmanogepix against *Scedosporium* spp. (*n* = 12 cumulative isolates) show promise and require further evaluation (53–55). Importantly, ongoing assessment of the correlation between *in vitro* data, host status, and clinical outcomes is crucial.

### Conclusion

Our data indicate that species-specific and inter-species differences exist in the distribution of antifungal susceptibility patterns among *Scedosporium* and *L. prolificans*. Although the number of rare *Scedopsorium* species was limited, our *in vitro* results suggest that OLF may be a promising therapeutic agent for these organisms. Additionally, our clinical data highlight that host status, along with effective antifungal therapy, plays a crucial role in determining treatment outcomes.

## SUPPLEMENTAL MATERIAL

Table S1 jcm.01550-24-s0001.xlsx The MIC/MEC distribution of 103 clinical *Scedosporium* spp. and *Lomentospora prolificans* strains to eight antifungal agents.

Table S2 jcm.01550-24-s0002.doc Densities of the conidial suspensions used for antifungal susceptibility testing, concentrations of antifungals tested, and incubation times for determining MIC (all except MFG) and MEC (only MFG).

ASM does not own the copyrights to Supplemental Material that may be linked to, or accessed through, an article. The authors have granted ASM a non-exclusive, world-wide license to publish the Supplemental Material files. Please contact the corresponding author directly for reuse.
