# Supplementary material for: Clinical significance and antifungal susceptibility profile of 103 clinical isolates of Scedosporium species complex and Lomentospora prolificans obtained from NIH patients
Source: J Clin Microbiol. 2025 Mar 7;63(4):e01550-24. doi: 10.1128/jcm.01550-24 (PMC11980389; doi:10.1128/jcm.01550-24)
Supplement: Table S2 — Densities of the conidial suspensions used for antifungal susceptibility testing, concentrations of antifungals tested, and incubation times for determining MIC (all except MFG) and MEC (only MFG). [file jcm.01550-24-s0002.doc]

**Supplementary table2:** Densities of the conidial suspensions used for antifungal susceptibility testing, concentrations of antifungals tested, and incubation times for determining MIC (all except MFG), and MEC (only MFG).

|  | **Densities of the conidial suspensions inoculated (adjusted to an absorbance at 530 nm)** | **Number of Concentrations Tested** | **Incubation times for determining MIC /MEC, hours** |
| --- | --- | --- | --- |
| **AmB - amphotericin B** | 0.15 to 0.17 | 0.016 to 16 µg/mL | 72 |
| **ITC – itraconazole** | 0.15 to 0.17 | 0.016 to 16 µg/mL | 72 |
| **VRC – voriconazole** | 0.15 to 0.17 | 0.016 to 16 µg/mL | 72 |
| **POSA – posaconazole** | 0.15 to 0.17 | 0.016 to 16 µg/mL | 72 |
| **ISA – isavuconazole** | 0.15 to 0.17 | 0.016 to 16 µg/mL | 72 |
| **TRB – terbinafine** | 0.15 to 0.17 | 0.016 to 16 µg/mL | 72 |
| **MFG – micafungin** | 0.15 to 0.17 | 0.016 to 16 µg/mL | 48 |
| **OLF – olorofim** | 0.15 to 0.17 | 0.016 to 16 µg/mL | 72 |
